# Small noncoding RNA modulates japanese encephalitis virus replication and translation in *trans*

**DOI:** 10.1186/1743-422X-8-492

**Published:** 2011-11-01

**Authors:** Yi-Hsin Fan, Muthukumar Nadar, Chiu-Chin Chen, Chia-Chen Weng, Yun-Tong Lin, Ruey-Yi Chang

**Affiliations:** 1 Department of Life Science and Institute of Biotechnology, National Dong Hwa University, Hualien 97401, Taiwan

## Abstract

**Background:**

Sequence and structural elements in the 3'-untranslated region (UTR) of Japanese encephalitis virus (JEV) are known to regulate translation and replication. We previously reported an abundant accumulation of small subgenomic flaviviral RNA (sfRNA) which is collinear with the highly conserved regions of the 3'-UTR in JEV-infected cells. However, function of the sfRNA in JEV life cycle remains unknown.

**Results:**

Northern blot and real-time RT-PCR analyses indicated that the sfRNA becomes apparent at the time point at which minus-strand RNA (antigenome) reaches a plateau suggesting a role for sfRNA in the regulation of antigenome synthesis. Transfection of minus-sense sfRNA into JEV-infected cells, in order to counter the effects of plus-sense sfRNA, resulted in higher levels of antigenome suggesting that the presence of the sfRNA inhibits antigenome synthesis. *Trans*-acting effect of sfRNA on JEV translation was studied using a reporter mRNA containing the luciferase gene fused to partial coding regions of JEV and flanked by the respective JEV UTRs. *In vivo *and *in vitro *translation revealed that sfRNA inhibited JEV translation.

**Conclusions:**

Our results indicate that sfRNA modulates viral translation and replication *in trans*.

## Background

Japanese encephalitis virus (JEV), a member of the *Flaviviridae *family, is a major zoonotic agent. Pigs and birds are the principal viremic hosts and mosquitoes are responsible for the transmission between these vertebrates to human [1]. In humans, JEV causes acute meningomyeloencephalitis with high mortality rate [2]. The JEV genome is a single-stranded positive sense RNA of about 10,976-nts that encodes a single large open reading frame (ORF) flanked by a 95-nucleotide (nt) long 5' untranslated region (UTR) and a 585-nt long 3' UTR with no poly A tail. Cap-dependent translation of the JEV ORF results in a polyprotein which is co- and post-translationally processed by viral as wells as host proteases to yield three structural proteins (C, prM, and E), and seven nonstructural proteins (NS1, NS2A, NS2B, NS3, NS4A, NS4B and NS5)[3].

As with all positive-sense RNA viruses, JEV RNA replication begins with the synthesis of negative-strand antigenome, which serves as a template for the synthesis of progeny positive-strand genomic RNA. The asymmetric RNA replication leading to 10- to 100-fold excess of positive strands over negative strands which was observed in Kunjin virus and dengue virus (DENV), and in JEV infected cells [4-7]. In addition to genome and antigenome, flaviviruses produce a small subgenomic RNA (named sfRNA) representing highly conserved regions of the 3'-UTR [8-11]. The sfRNA is more abundant in JEV-infected mosquito cells than mammalian cells and the molar ratio of sfRNA to genomic RNA can range from 0.25 to 5.14 [8]. The abundant accumulation of this RNA suggests that it may play an important role in viral life cycle. Using West Nile virus (WNV) as a model, Pijlman et al. demonstrated that the sfRNA is a product of incomplete degradation of viral genome by cellular ribonuclease XRN1 and is essential for virus-induced pathogenicity [11]. It was reported that a pseudoknot structure at the 3'-UTR is responsible for stalling XRN1 from degrading the RNA further, which results in sfRNA [9,10].

The 3'-UTR of flaviviruses has been shown to serve various important functions such as translation, replication, and encapsidation [12-17]. There are several functional motifs in the 3'-UTR including the conserved sequences (CS motifs), cyclization motifs, pseudoknot structure, and the 3'-stemloop (3'-SL) motif. Two pairs of cyclization motifs were reported. Hahn et al. first reported that the 5' and 3' conserved sequences are complementary to each other and potentially form a cyclization structure [18]. The second pair of cyclization motif is the upstream of AUG codon (5'UAR), which is complementary to the sequences located in the 3'-UTR (3'UAR). The UAR cyclization motifs have been shown to be required for replication in DENV and WNV [19-23]. In addition, Friebe and Harris identified another element located downstream of the AUG (designated 5'DAR) also involved in DENV replication and possibly genome cyclization [24]. Although these conserved sequences were found in JEV, the functions of these motifs have not been characterized in detail. Yun et al. analyzed the 3'-UTR of JEV and defined it into six domains [25]. By constructing serial deletion mutants, they demonstrated that the two 3'-proximal domains are sufficient for RNA replication, while the other four domains are dispensable but required for maximal replication efficiency suggesting the *cis*-acting sequences required for JEV replication might be slightly different from those other flaviviruses. In addition to RNA-RNA interactions, numerous studies have been shown that 3'-UTR interacts with both viral and cellular proteins, and is required for RNA synthesis and translation [15,26-36]. In JEV, viral NS3 and NS5 proteins as well as cellular Mov 34 and La proteins have been shown to bind to the 3'-SL and play roles in viral replication [37-39]. Previously we showed that the cellular protein GAPDH binds more efficiently to the 3' end of minus-strand RNA than to the 3'-SL of plus-strand RNA, suggesting a role for promoting asymmetric RNA replication [40].

The presence of such essential motifs in sfRNA and its abundance in infected cells present a compelling indication of a possible function that prompted us to elucidate its role in the viral life cycle at the cellular level. We found that high levels of sfRNA accumulates in the cytoplasm during the late stages of viral life cycle suggesting that sfRNA may inhibit either viral translation or minus-strand synthesis or both. To test this, plus- or minus-strand forms of the sfRNA were separately transfected in virus-infected cells and the effects on genome and antigenome accumulation were measured. The effect of sfRNA on JEV translation was determined by co-transfecting plus or minus sense of sfRNA with a luciferase reporter RNA for *in vivo *translation studies in cultured cells and a rabbit reticulocyte lysate assay system was used for *in vitro *translation studies. Our results indicated that the sfRNA inhibits antigenome synthesis and also down regulates viral translation *in trans*.

## Results

### sfRNA localizes to the cytoplasm along with a major proportion of genomic RNA while a small proportion of genomic RNA is localized in the nucleus

**Figure 1:**
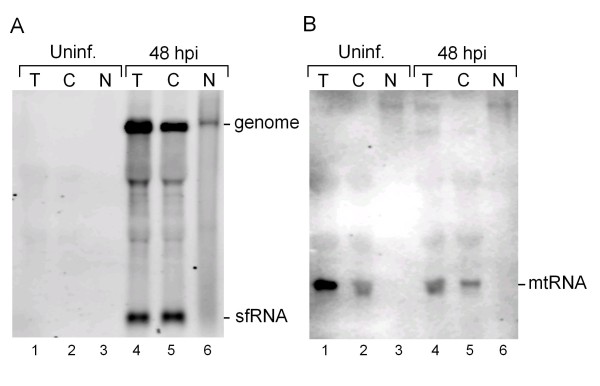
**Northern analysis of cytoplasmic and nuclear RNA fraction**. RNA was extracted from uninfected BHK-21 cells (uninf.) and from cells infected with JEV at MOI of 0.1 at 48 h postinfection. Northern analysis was performed using DIG-labeled oligonucleotides complementary to nts 10,950-10,976 in the 3'-UTR (A) or to mitochondria RNA (mtRNA) (B). T, total RNA extract; C, cytoplasmic fraction, and N, nuclear faction.

Replication of flavivirus RNA takes place mainly in the cytoplasm, however, the major replicase proteins NS3 and NS5 were also found to localize within the nucleus [41]. To determine subcellular distribution of the sfRNA, which is important toward to understanding its function, total RNA from the nuclear and cytoplasmic fractions from both uninfected and infected BHK-21 cells were subjected to Northern blot analysis using 3JEV10950(-) oligonucleotide probe that detects genome and the sfRNA (Figure 1A). The results showed that the sfRNA and the genomic RNA could be detected in the total RNA extract and the cytoplasmic fraction (Figure 1A, lanes 4 and 5). In the nuclear fraction, only a few genomic RNA was detected, whereas the sfRNA was not detected at all (Figure 1A, lane 6). These signals were not detected in any of the fractions of the uninfected cells (Figure 1A, lanes 1 to 3). Mitochondrial 12S rRNA was used as a cytoplasm specific subcellular marker to exclude the possibility of the JEV genomic RNA detected in the nuclear extract coming from cytoplasmic contamination during fractionation. As shown in Figure 1B, the 12S rRNA was detected in the total RNA extract and cytoplasmic fraction (lanes 1, 2, 4, 5) but not in the nuclear fraction (lanes 3, 6), indicating that the subcellular fractionation of nucleus versus cytoplasm was accurate and efficient.

### Time course study of viral genome and antigenome synthesis in JEV-infected cells

**Figure 2:**
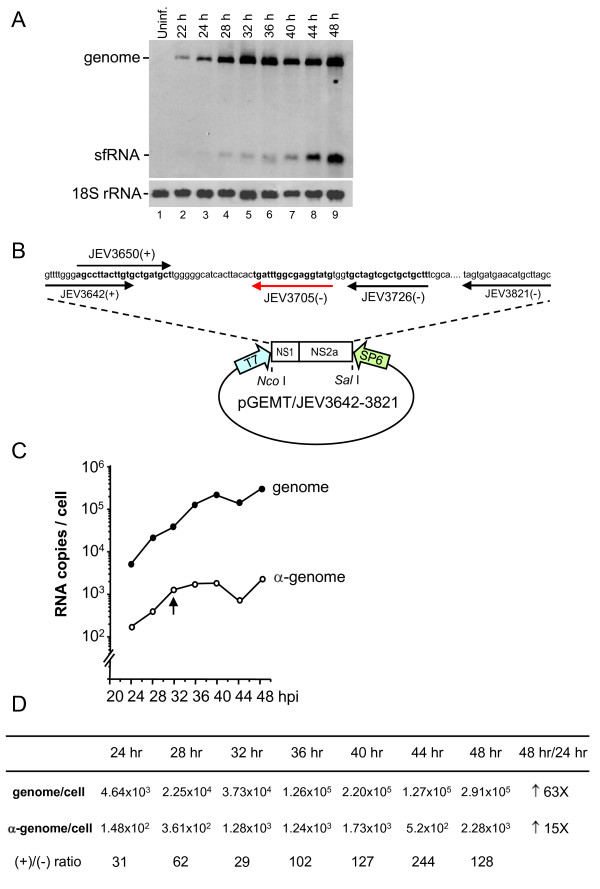
**Kinetics of genomic and antigenomic RNA synthesis in JEV-infected BHK-21 cells**. A. BHK-21 cells were infected with JEV at an MOI of 0.01, and cytoplasmic RNAs were extracted at the indicated time points postinfection and were subjected to Northern analysis as described in Fig.1. Oligonucleotide probe detecting 18S rRNA is shown at the bottom. B. Diagram of primer positions and the vector used for generating RNA transcripts as standards for real-time RT-PCR assay. C. The RNAs were subjected to real-time RT-PCR assay. Amounts of genome and antigenome per cell were plotted and calculated (C and D). Vertical arrow in panel C indicates the time when sfRNA becomes apparent.

To determine the appearance of the sfRNA with the kinetics of genome and antigenome, cells were infected with JEV at an multiplicity of infection (MOI) of 0.01, the RNA was extracted at 4 h intervals during a 48-h postinfection period and analyzed by Northern blot. The probe used was 3JEV10950(-) oligonucleotide which detects RNA containing the very 3'-terminal 27 nts of the JEV genome. The results showed that genomic RNA was detected at 22 h and sfRNA at 28 h postinfection, and their abundance continued to increase throughout the experimental period (48 h; Figure 2A). To more precisely determine the kinetics of genome and antigenome accumulation in JEV-infected cells in context of sfRNA, cytoplasmic RNAs extracted at the indicated time points were subjected to one-step real-time RT-PCR. Sequence specific primer designed for the genome or antigenome was used during RT step (Figure 2B). A linear standard curve obtained from known input RNA copies to the threshold cycle (Ct value) in the real-time RT-PCR assay was determined (data not shown). The amount of intracellular genome or antigenome per cell was calculated by dividing the copy number of genomic or antigenomic RNA by the number of cells counted at each time point. As shown in Figure 2C and 2D, the genomic RNA was 4.64 × 10^3 ^copies per cell at 24 h postinfection, and its abundance continued to increase throughout the 48-h infection period, except for a slight diminution at 44 h postinfection. The RNA levels reached to 2.91 × 10^5 ^copies per cell at 48 h postinfection. The accumulation of the antigenome followed the same trend as the genomic RNA throughout the experimental period but the amount was less by one to two orders of magnitude than that of genomic RNA. From 24 to 48 h postinfection there was a 63-fold increase in genome accumulation whereas only a 15-fold increase for the antigenome indicating that the increasing rate of genomic RNA accumulation is nearly four times faster than that of the antigenomic RNAs (Figure 2D). Interestingly, as shown in Northern analysis the sfRNA becomes apparent at the time point when antigenome reaches a plateau suggesting a role for sfRNA in the regulation of minus strand synthesis.

### The presence of the sfRNA inhibits antigenome synthesis

**Figure 3:**
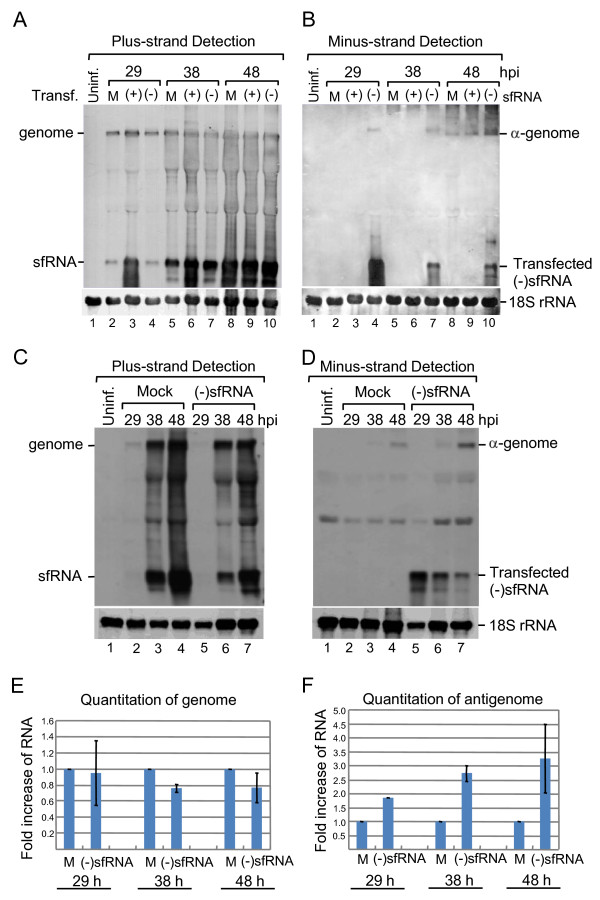
**Effect of (+)sfRNA and (-)sfRNA on JEV RNA synthesis, when transfected into JEV-infected BHK-21 cells**. A and B. Cells were either left uninfected (uninf., lane 1), or infected (lanes 2-10) with JEV at an MOI of 0.01. Plus-sense (+) (lanes 3, 6 and 9) and minus-sense (-) (lanes 4, 7 and 10) of sfRNAs were transfected at 28 hpi, or mock transfected (lanes 2, 5 and 8). Cytoplasmic RNA was extracted at the indicated hours post-infection (hpi). RNA was probed with a DIG-labeled minus-sense sfRNA to detect plus strands (A) or with plus-sense sfRNA to detect minus-strands (B). Oligonucleotide probe detecting 18S rRNA is shown at the bottom. C and D. Cells were either left uninfected (uninf., lane 1), or infected (lanes 2-7) with JEV at an MOI of 0.01. Minus-sense of sfRNAs (lanes 5-7) were transfected at 28 h postinfection (hpi), or mock transfected (lanes 2-4). Cytoplasmic RNA was extracted at the indicated time point. Dig-labeled riboprobes were used as indicated at the top. Oligonucleotide probe detecting 18S rRNA is shown at the bottom. Effect of transfecting (-)sfRNA into the JEV-infected BHK-21 cells on genome (E) or antigenome (F) was plotted. Error bars indicate the standard deviations of results from three independent experiments.

To elucidate the possible function of the sfRNA during viral replication, plus- and minus-strand forms of the sfRNA was separately transfected in virus-infected cells and the effects on genomic and antigenomic accumulation were measured. The intention for the transfection of minus-strand sfRNA into JEV-infected cells was to counter the effects of the naturally occurring plus-strand sfRNA and observe the outcome. BHK-21 cells were infected with JEV at an MOI of 0.01 and *in vitro *transcribed plus- or minus-strand forms of the sfRNA were transfected separately at 28 h postinfection, at which time point the sfRNA was detected by Northern blot in mammalian cells (Figure 2A, lane 4). Cytoplasmic RNAs were extracted and examined with Northern blotting using *in vitro *transcribed DIG-labeled riboprobes to detect plus- and minus-strands, respectively. The results showed that transfection of the (+)sfRNA did not affect genomic or antigenomic RNA accumulation compared to mock transfection (Figure 3A and 3B, lanes 3, 6, 9), while transfecting of the (-)sfRNA increased amount of antigenome synthesis at 48 h postinfection (Figure 3B, lane 10). Interestingly, an RNA band slightly higher than the antigenome was observed when transfecting of (-)sfRNA (Figure 3B, lanes 4, 7, 10). This higher molecular weight molecule was not detected when transfecting with (+)sfRNA or mock transfected cells. The input (-)sfRNA was transcribed from SP6 promoter of the *Nco *I-linearized pGEMT/JEV10450-10976 resulting in extra 75 nts at the 5'-end and 15 nts at the 3' end derived from vector sequences. These extra sequences could have primed cellular sequences during the amplification step resulting in an unexpected band. To generate a (-)sfRNA with precise sequences complementary to the authentic sfRNA, we then constructed pUC18/JEV(-)10976-10454 and used for generating (-)sfRNA without extra sequences at the termini. Transfecting of the (-)sfRNA did not have significant influence on the accumulation of genome (Figure 3C), while the amount of antigenome increased compared to mock transfection (Figure 3D). These experiments were repeated at least three times and the amount of RNA on each lane was quantitated by densitometry, normalized to 18S rRNA, and compared to mock transfection (Figure 3E and 3F). The results showed that transfection of (-)sfRNA did counter the effects of sfRNA resulting in higher levels of antigenome (Figure 3D and 3F). These results suggest that the presence of the sfRNA plays a role in the inhibition of antigenome synthesis.

### The sfRNA inhibits translation

**Figure 4:**
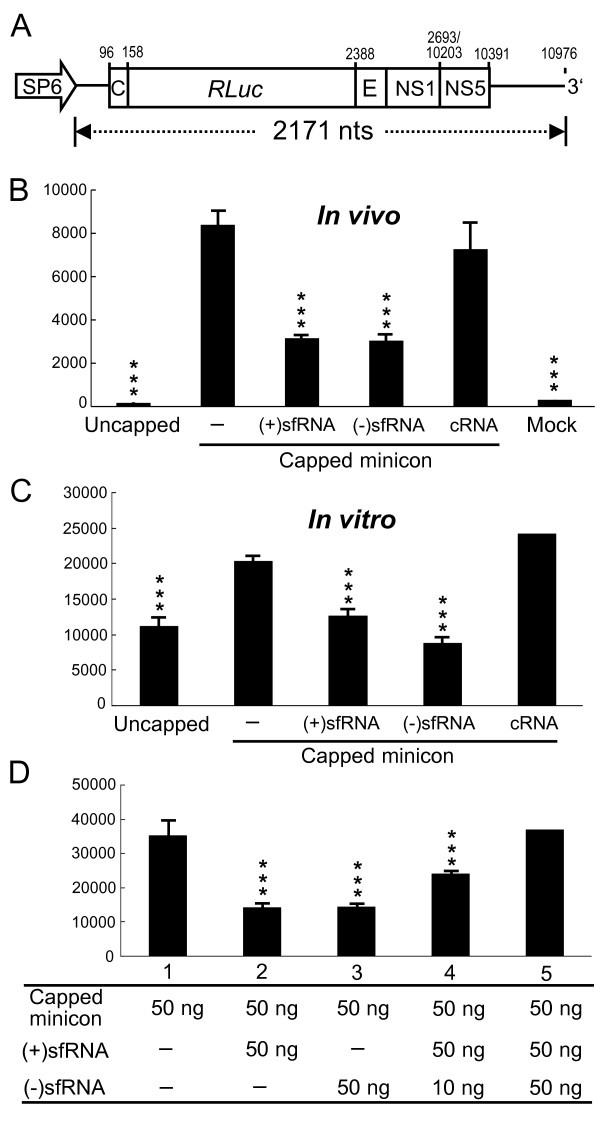
**Effect of sfRNA on viral translation in cultured cells and *in vitro***. A. A diagram of the JEV minicon RNA is shown. B. JEV minicon RNAs were transcribed *in vitro *without or with capped analog and then transfected alone (-) or cotransfected with (+)sfRNA, (-)sfRNA, or control RNA (cRNA). The *Renilla *luciferase activity of cell lysates was determined at 8 h posttransfection. C. *Renilla *luciferase assays of JEV minicon RNA translated in rabbit reticulocyte lysate in the presence of (+)sfRNA, (-)sfRNA or control RNA. D. The effect of different amounts of (+)sfRNA or (-)sfRNA on *in vitro *translation of minicon RNA in rabbit reticulocyte lysate. The *Renilla *luciferase activity of cell lysate with five different treatments is shown. *** represents *P *< 0.005 in comparison with capped JEV minicon only.

It has been shown that the 3'-UTR possesses many highly ordered secondary structures involved in viral translation most of which are also present in sfRNA. To test whether the presence of sfRNA affects viral translation, a JEV minicon containing *Renilla *luciferase reporter gene constructed with authentic JEV 5' and 3' UTRs and part of the coding sequences (Figure 4A; described in Materials and Methods) was used. *In vitro *transcribed minicon RNA was cotransfected with either (+)sfRNA, (-)sfRNA, or control RNA (cRNA) and effects on reporter translation were measured at 8 h posttransfection. Interestingly, transfecting either (+)sfRNA or (-)sfRNA but not control RNA inhibited luciferase translation driven by JEV 5' UTR (Figure 4B). Consistent with *in vivo *translation assay, *in vitro *translation of luciferase was inhibited by the addition of (+)sfRNA as well as (-)sfRNA in rabbit reticulocyte lysate (Figure 4C). In order to confirm if the (-)sfRNA could counter the effects of (+)sfRNA, both sense and antisense sfRNA were added simultaneously into the *in vitro *translation mix to measure their effect on minicon translation. As shown in Figure 4D, the inhibitory effect of (+)sfRNA on translation was rescued by the addition of (-)sfRNA. When equimolar amounts of plus-sense and minus-sense sfRNA were added to the reaction, translation was restored to control (minicon only) levels.

## Discussion

The discovery of sfRNA in flaviviruses has generated considerable interest in its generation, localization, and its possible function in flavivirus infected cells. In this study, we have shown that JEV sfRNA is localized in cytoplasm along with the genomic RNA (Figure 1A). Although the biogenesis of JEV sfRNA has not yet been studied, it has been reported that in WNV and YFV, sfRNA is a product of incomplete degradation of viral genome by cellular ribonuclease XRN1 and is co-localized to the P-body in the cytoplasm [11]. Consistent with their report we found that JEV sfRNA is also localized to the cytoplasm along with the genomic RNA. On the other hand, we also found that a few genomic RNA is also localized to the nucleus consistent with reports on the presence of flaviviral proteins and flaviviral RNA replication in the nuclear fraction [41-43]. The presence of abundant sfRNA in the cytoplasm of infected cells hints at possible roles of the sfRNA in cytoplasmic events during infection, namely viral RNA replication and translation.

The RNA synthesis of plus-strand RNA viruses is asymmetrical meaning that positive-sense genomic RNA strands are generated in excess over minus sense antigenome and the ratio is about 10:1 to 100:1. Northern analysis from a previous study showed that the ratio of plus-to-minus strands at 8 h postinfection was 3:1 which rapidly increased thereafter to 11.7:1 by 18 h postinfection in porcine kidney cells infected with JEV at an MOI of 10 [7]. In this study, we describe the kinetics on the synthesis of JEV genome and antigenome in BHK-21 cells at an MOI of 0.01 using strand specific oligonucleotides for real-time RT-PCR. Our results indicated that the ratio of plus-to-minus strands during 24-48 h postinfection was in the range of 29:1 to 244:1 during inspection period (Figure 2D). Interestingly, we found that the time point at which the antigenome accumulation reaches a plateau coincides with the appearance of sfRNA as shown in our results from Northern and real-time RT-PCR analyses. It would be ideal to show the time course of antigenome and sfRNA accumulation together but since it was impossible to distinguish sfRNA accumulation from genomic RNA accumulation in real-time RT-PCR experiments, we employed Northern analysis which clearly distinguishes the two (Figure 2A). The time course of antigenome, on the other hand, real-time RT-PCR is much more sensitive than Northern analysis especially during the early time points (Figure 2C). Thus, we compare the same amounts of RNA under the same condition by these two different methods.

The artificial addition of (-)sfRNA (by transfection) at 28 h postinfection countered the effects of naturally occurring sfRNA thereby increasing the accumulation of antigenome (Figure 3D and 3F) indicating that sfRNA could negatively interfere with antigenome synthesis. The addition of (-)sfRNA may not only anneal to the naturally occurring plus-sense sfRNA but also to the 3'-UTR of the genomic RNA. However, the probability of the minus-sense sfRNA to bind to the plus-sense sfRNA is more than its binding to the genomic 3'-UTR because of (i) the binding strength of same size shorter complementary RNA should be greater than the binding of a short RNA sequence to a large RNA polynucleotide akin to a highly complementary primer dimer and a PCR template. This was observed in our *in vitro *luciferase assays (Figure 4) where the individual addition of either only (+)sfRNA or (-)sfRNA reduced translation but the addition of both (+)sfRNA (50 ng) and (-)sfRNA (10 ng) into the reaction partially restored translation and the addition of more (-)sfRNA (50 ng) restored translation to control levels probably because the plus-sense and the minus-sense sfRNA hybridized to form duplex RNA thereby preventing sfRNA from interfering with translation; (ii) cyclization of the genome could render the 3'-UTR inaccessible to the minus-sense sfRNA. Curiously, when (-)sfRNA is transfected, the amount of naturally occurring plus-sense sfRNA does not decrease (Figure 3A and 3C) and is similar to that of mock, indicating that naturally occurring plus-sense sfRNA is either not degraded or that the rate of sfRNA generation (RNA turnover) is very high.

**Figure 5:**
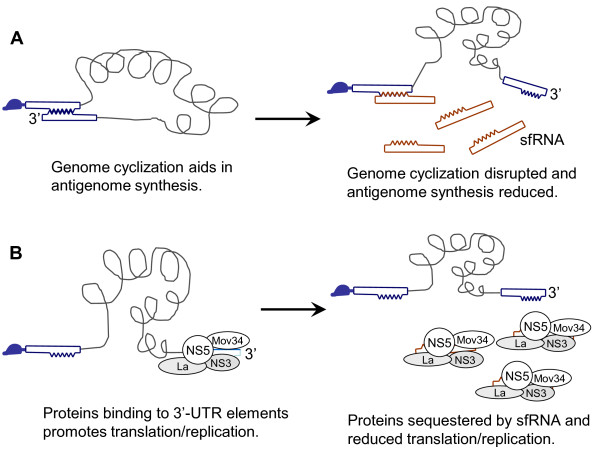
**Model depicting the role of sfRNA in flaviviral RNA replication and translation**. A. The diagrammatic representation of flaviviral cyclization, through the cyclization elements, which promote antigenome synthesis. The sfRNA disrupts genome cyclization by interacting with the 5' cyclization elements and thereby reduces antigenome synthesis. B. Flaviviral genome contains protein-binding elements in the 3'-UTR. Viral and host proteins bind to their respective RNA elements in the 3'-UTR and promote translation and replication. The presence of sfRNA sequesters most of the proteins reducing its availability for flaviviral translation and replication.

The presences of *cis*-acting sequences including promoters, enhancers, and repressors that aid in the in regulation of minus-strand synthesis have been reported in many plus-strand RNA viruses [44-47] and these elements may consequently contribute to asymmetrical RNA synthesis. Viral or host proteins may also contribute to asymmetric replication *in trans *[48-50]. Several RNA motifs within 5' and 3'-UTR, for instance, 5'-CS/CS1, 5'-UAR/3'-UAR, and 5'DAR/3'DAR are involved in RNA-RNA interactions. These RNA-RNA interactions have been demonstrated to be required for viral replication. In DENV, a stemloop A (SLA) has been identified at 5' end of the genome which was shown to be required for long-range RNA-RNA interaction and the recruitment of virus RdRp which is then transferred to the initiation site present in the 3'-UTR in order to promote minus-strand RNA synthesis [51]. In addition, the balance between circular and linear forms of the DENV genome is crucial for viral replication [52]. Thus, the presumable mechanism of the suppression of antigenome synthesis by JEV sfRNA may be due to the interruption of genome cyclization by its complementarity to the 5'-end elements of JEV genome (Figure 5A). Since the sfRNA is in high molar excess it could also be assumed that free sfRNA (sfRNA not bound to the 5' of JEV genome) could further reduce antigenome synthesis through a second mechanism by competing for viral and host proteins that would otherwise bind to the 3' UTR and promote antigenome synthesis (Figure 5B). We hypothesis that sfRNA through its *trans*-acting function could be one of the factors contributing to the asymmetry in JEV RNA replication.

It has also been reported that the RNA elements in the flaviviral 3'-UTR influences viral translation efficiency [13,15,33,35]. However, Alvarez et al. developed a replicon system that can be used to discriminate between translation and RNA replication. They demonstrated that deletion of individual domains of the 3'-UTR did not significantly affect viral translation but it impaired or abolished RNA synthesis [12]. Our results showed that JEV translation efficiency in cultured cells was reduced in the presence of sfRNA (Figure 4). JEV translation efficiency *in vitro *was also impaired in the presence of sfRNA but was restored to control levels by the addition of equal amounts antisense sfRNA into the reaction. This clearly shows that sfRNA does impair JEV translation *in tran*s, as depicted in Figure 5B, proteins binding to 3'-UTR elements are essential to promote viral translation. Transfection of (+)sfRNA sequesters proteins binding to the 3'-UTR of genome and reduces translation, while if (-)sfRNA is transfected it could bind to the 3'-UTR of the genome and prevent the host proteins from binding the 3'UTR (competes with host factors binding to the 3'UTR). In addition, transfection of (-)sfRNA could also prevent the interaction of the 3' and 5' regions of the genome and that too could reduce translation. Thus, transfecting of either (+) or (-)sfRNA reduces translation. Furthermore, the sfRNA could titrate the host factors and even the newly synthesized viral proteins like RdRp thereby drastically reducing minus-strand RNA synthesis that results in the aforementioned asymmetry in RNA accumulation.

## Conclusions

As seen in our results (Figures 2A, 3A and 3C), sfRNA is present in great abundance in the late stages of the viral replication cycle and that sfRNA interferes and impairs both antigenome synthesis and JEV translation (Figures 3 and 4). It could be thought that the JEV genomic RNA produced during the late stages of the viral replication cycle is bound for packaging and should not be used as templates for antigenome synthesis or for translation and the presence of sfRNA is suspected to compete against the translation and antigenome synthesis. From our data, we conclude that sfRNA could be the switch (a *trans*-acting riboswitch) that shuts down both antigenome synthesis and JEV translation thereby promoting only genomic RNA synthesis that needs to be packaged and released for the next infectious cycle.

## Materials and methods

### Cells and viruses

Baby hamster kidney (BHK-21) cells were grown in RPMI 1640 medium supplemented with 2% fetal bovine serum (FBS) (Gibco-BRL) at 37°C. JEV strain RP9, a variant of NT109 isolated originally from *Culex tritaeniorhynchus *was used in this study [53].

### Construction of plasmids

pGEMT/JEV3642-3821 plasmid (Figure 2B) used for making RNA standard in real-time RT-PCR was generated by cloning the 180-nt PCR product amplified from JEV cDNA with the JEV3642(+) (nt 3642-3662) and JEV3821(-) (nt 3802-3821) primers into pGEMT-easy vector (Promega). To generate pGEMT/JEV10450-10976 construct, we followed the same method as for pGEMT/JEV3642-3821 plasmid, except that JEV10450(-) (nt 10450-10476) and JEV10950(+) (nt 10950-10976) primers were used for PCR. PCR products were amplified from sfRNA with primers containing T7 promoter at 5' end and a unique restriction site at the 3' end, then cloned into pUC18 plasmid to generate pUC18/JEV(+)10450-10976, and pUC18/JEV(-)10976-10454 for making (+)sfRNA and (-)sfRNA respectively. Sequences of each construct were confirmed by sequencing.

### RNA preparation and Northern blot analysis

RNA extraction and Northern analyses were done as described previously [8]. Briefly, total RNA was extracted with Trizol (Invitrogen) or REzol™ C&T reagent (Protech). Cytoplasm/Nucleus fractionation was done using Cytoplasmic & Nuclear RNA Purification kit (Norgen) according to manufacturer's instruction. Approximate 2.5 μg or 7-10 μg of cytoplasmic RNA were used per lane in formaldehyde-agarose gel electrophoresis for the detection of plus- or minus-strand, respectively. To label oligonucleotide probe, approximately 100-pmol of oligonucleotide was 3' tailed with Digoxigenin (DIG)-ddUTP using a DIG Oligonucleotide 3'-End Labeling Kit (Roche Molecular Biochemicals). For labeling sfRNA probe, 1 μg of linearized DNA was used for *in vitro *transcription with DIG RNA labeling mix (Roche Molecular Biochemicals). Hybridization was done at 54°C for oligonucleotide probes and 68°C for riboprobes. DIG luminescent detection of the viral specific bands was done according to the manufacturer's instructions (Roche Molecular Biochemicals).

### Synthetic oligonucleotides and accession number

JEV genome nucleotide positions correspond to those for JEV RP9, GenBank accession number AF014161. 3JEV10950(-) oligonucleotide (5'-AGATCCTGTGTTCTTCCTCACCACCAG-3') detects RNA containing the very 3'-terminal 27 nts of the JEV genome. 18S rRNA(-) oligonucleotide (5'-GCACTTACTGGGAATTCCTCG-3') location corresponds to mouse 18S rRNA, GenBank accession number X00686. Mitochondria 12S rRNA(-) oligonucleotide (5'-AAGGCCAGGACCAACCT-3') was synthesized according to GenBank accession number NC_005089.

### Real-time RT-PCR

The method used for real-time RT-PCR assay was as described previously [54]. The *in vitro *transcripts of positive-sense RNA were generated from T7 transcription (Promega) of the *Sal *I-linearized pGEMT/JEV3642-3821 and the minus-sense transcripts were transcribed from SP6 promoter of the *Nco *I-linearized pGEMT/JEV3642-3821 (as diagramed in Figure 2B). The amount of purified RNA was measured by spectrophotometry and the copy number was calculated based on the concentration measured and its molecular weight. The known amounts of RNAs were serially diluted 10-fold (1.78 × 10^11 ^to 1.78 × 10^5 ^copies) and subjected to real-time RT-PCR using the one-step RT-PCR master mix reagent kit following the manufacturer's instructions (Applied Biosystems). Oligonucleotides JEV3642(+) and JEV3821(-) were used as primers for binding to minus and plus-strand RNA, respectively, during RT step carried out at 48°C for 30 min. The PCR amplification conditions were 95°C for 10 min, followed by 40 cycles of 95°C for 15 sec and 60°C for 1 min with primers JEV3650(+), JEV3726(-) and TaqMan probe JEV3705(-) (sequences and binding positions are illustrated in Figure 2B). The assay was performed on an ABI 7000 Sequence Detector using TaqMan One-Step RT-PCR master mix to analyze the emitted fluorescence during amplification (Applied Biosystems). A linear equation of known amounts of RNA to CT value was determined.

The intracellular plus- or minus-strand RNAs in JEV-infected cells were determined by using strand specific primers during RT step as described above. Cytoplasmic RNAs were extracted at the indicated time points postinfection. RNA from each time point was diluted to a concentration of 100 ng/μl and 10 ng/μl, respectively, and subjected to real-time RT-PCR together with the known amount of *in vitro *transcripts. The amount of intracellular genome or antigenome per cell was determined by dividing the copy number by the numbers of cells counted at each time point postinfection.

### RNA transfection

For run-off transcription, *Sal *I-linearized pUC18/JEV10450-10976 or *Xba *I-linearized pUC18/JEV(-)10976-10454 were used for making plus- or minus-strand form of the sfRNA, respectively. Before RNA transfection, cells in 6-well plates at 50 to 80% confluence (approximately 2 × 10^6 ^cells) were infected with JEV RP9 at an MOI of 0.01 by incubating cells with inoculum at 37°C for 1 h, refeeding with 2 ml of growth medium containing 5% FBS, and incubating at 37°C for 27 h. For transfection, each dish of cells was rinsed three times with RPMI and treated for 10 min at 0°C with 200 μl of Opti-MEM medium (Gibco-BRL) containing 10 μl of lipofectin (Invitrogen) and 1 μg of RNA transcripts. Cells were rinsed with 2 ml of RPMI medium three times and incubated at 37°C with 2 ml of medium containing 5% FBS until cytoplasmic RNA extraction was done at the indicated time points.

### Luciferase assay

JEV minicon (kindly provided by Dr. Yi-Ling Lin) contains a *Renilla *luciferase (Rluc) fused in-frame to the JEV coding regions as a single ORF in the following order; the core (nt 96-158), Rluc (933 nts), E (nt 2388-2477), NS1 (nt 2478-2693), and NS5 (nt 10203-10391), and this ORF unit was flanked by the authentic JEV 5'- and 3'-UTRs (Figure 4A). The Rluc-reporter plasmid was transcribed using a Megascript SP6 Transcription kit (Ambion) according to the manufacturer's instructions, in the presence or absence of ^m^7GpppA nucleotide (New England Biolabs). Luciferase assays were performed using extracts from transfected cells and also from the *in vitro *translation assay. For *in vivo *translation in cultured cells, 1 μg of transcribed minicon RNA, together with 1 μg of plus- or minus-strand of sfRNA were transfected into BHK-21 cells using Lipofectamin 2000 (Invitrogen). *Renilla *luciferase activity was measured at 8 hour posttransfection. For *in vitro *translation, 50 ng of RNAs were translated in nuclease-treated rabbit reticulocyte lysate (Promega) in the presence of 40 units of RNasin Ribonuclease Inhibitor (Promega), 20 μM of each amino acid, and either plus-strand, minus-strand of sfRNA, or a 445-nt control RNA composed of JEV sequences (nt 2401-2689) plus 156 nts derived from vector sequences. The reactions were incubated at 30°C for 30 min and 2.5 μl of reaction sample was measured with 20/20^n ^Single-Tube Luminometer (Promega).

### Statistical analysis

For statistical analysis, one-way ANOVA Dunnet's multiple comparisons test was used to compare the control group against others (GraphPad Software, San Diego, CA, USA).

## List of abbreviations

CS: conserved sequences; DAR: downstream of the AUG; DENV: dengue virus; JEV: Japanese encephalitis virus; MOI: multiplicity of infection; sfRNA: small flaviviral RNA; SLA: stemloop A; UAR: upstream of the AUG; UTR: untranslated region; WNV: West Nile virus.

## Competing interests

The authors declare that they have no competing interests.

## Authors' contributions

YHF carried out the translation experiments and statistical analyses. MN designed one of the translation experiments, the model, and participated in drafting of the manuscript. CCC and CCW carried out the replication studies. YTL carried out the nuclear and cytoplasmic fractionation and Northern blot analysis. RYC conceived of the study, participated in its design and coordination, and finalized the manuscript in its final form. All authors read and approved the final manuscript.
